# Adolescent Cancer Education (ACE) to increase adolescent and parent cancer awareness and communication: study protocol for a cluster randomised controlled trial

**DOI:** 10.1186/1745-6215-14-286

**Published:** 2013-09-08

**Authors:** Richard G Kyle, Iona Macmillan, Petra Rauchhaus, Ronan O’Carroll, Richard D Neal, Liz Forbat, Sally Haw, Gill Hubbard

**Affiliations:** 1 Cancer Care Research Centre, School of Nursing, Midwifery and Health, University of Stirling (Highland Campus), IV2 3JH Inverness, UK; 2 Teenage Cancer Trust, 93 Newman Street, W1T 3EZ London, UK; 3 College of Medicine, Dentistry and Nursing, University of Dundee, DD1 5EH Dundee, UK; 4 School of Natural Sciences, University of Stirling, FK9 4LA Stirling, UK; 5 North Wales Centre for Primary Care Research, Bangor University, LL13 7YP Bangor, UK; 6 Cancer Care Research Centre, School of Nursing, Midwifery and Health, University of Stirling, FK9 4LA, Stirling, UK; 7 School of Nursing, Midwifery and Health, University of Stirling, FK9 4LA Stirling, UK

## Abstract

**Background:**

Raising cancer awareness among adolescents has potential to increase their knowledge and confidence in identifying cancer symptoms and seeking timely medical help in adolescence and adulthood. Detecting cancer at an early stage is important because it reduces the risk of dying of some cancers and thereby contributes to improved cancer survival. Adolescents may also play an important role in increasing cancer communication within families. However, there are no randomised controlled trials (RCT) of the effectiveness of school-based educational interventions to increase adolescents’ cancer awareness, and little is known about the role of adolescents in the upward diffusion of cancer knowledge to parents/carers. The aim of this study is to determine the effectiveness of a school-based educational intervention to raise adolescent and parent cancer awareness and adolescent-parent cancer communication.

**Methods:**

The Adolescent Cancer Education (ACE) study is a school-based, cluster RCT. Twenty secondary schools in the area covered by Glasgow City Council will be recruited. Special schools for adolescents whose additional needs cannot be met in mainstream education are excluded. Schools are randomised to receive a presentation delivered by a Teenage Cancer Trust educator in Autumn 2013 (intervention group) or Spring 2014 following completion of six-month follow-up measures (control group). Participants will be students recruited at the end of their first year of secondary education (S1) (age 12 to 13 years) and one parent/carer for each student, of the student’s choice. The primary outcome is recognition of cancer symptoms two weeks post-intervention. Secondary outcomes are parents’ cancer awareness and adolescent-parent cancer communication. Outcomes will be assessed at baseline (when adolescents are in the final term of S1), two-week, and six-month follow-up (when adolescents are in S2, age 13 to 14 years). Differences in outcomes between trial arms will be tested using multiple regression methods, adjusted for clustering by school. An audit of cancer-related and health-promotion activity within the school curriculum and environment during the RCT will be conducted at six-month follow-up to contextualise the intervention effect.

**Discussion:**

Results from the ACE study will provide evidence about the public health effectiveness of a school-based intervention designed to increase adolescent and parent cancer awareness and adolescent-parent cancer communication.

**Trial registration:**

ISRCTN75542411

## Background

Each year in the UK around 2,000 teenagers and young adults (TYA) aged 15 to 24 and 1,600 children aged 0 to 14 are diagnosed with cancer, which constitutes 0.6% and 0.5% of all cancer registrations, respectively [1]. Leukaemia is the most common cancer in children of both sexes (30% of all childhood cancer registrations), and malignant melanoma is the most common cancer among female TYAs (17% of all female TYA registrations) and testicular cancer is the most common among male TYAs (27%) [1]. Diagnostic delay is perceived to be a problem for adolescents [2-4] and lack of awareness of cancer warning signs and symptoms may be factors for delay [5-8].

Improving cancer awareness during adolescence may equip young people with the knowledge and positive health-related and help-seeking behaviour for both adolescence and later life, as their risk of cancer increases with age. This is because detecting symptomatic cancer more quickly may increase the rates of survival and other positive cancer outcomes for some cancers [9,10], and improving public cancer awareness is one component of a comprehensive strategy to increase the proportion of people with earlier stage diagnosis [11,12]. Late detection is multi-factorial, but patient delay in visiting a General Practitioner (GP) may partially explain the problem [5,13,14] and lack of public awareness of cancer signs and symptoms may be further reasons for late diagnoses [6]. Hence, this protocol addresses the appraisal and help-seeking intervals of the diagnostic journey [5], and the methods and reporting are in keeping with recently published consensus guidelines [15].

We have completed pilot work of a school-based intervention that aimed to increase adolescent cancer awareness [16]. This intervention was an educational programme called ‘Let’s talk about it’ delivered by Teenage Cancer Trust in approximately 10% of UK schools each year (n = 600). ‘Let’s talk about it’ is a one-hour presentation delivered verbally by a single Teenage Cancer Trust educator to groups of adolescents in a classroom or assembly setting. Content is linked to outcomes from the ‘Health and Well-being’ section of the Curriculum for Excellence in Scotland [17] and Personal, Social, Health and Economic Education (PSHEE) in England and Wales [18]. Topics covered in the presentation included an introduction to cancer; identification of cancer warning signs; the physical, emotional and social impact of cancer; cancer diagnosis and treatment; and the importance of taking responsibility for your own health and well-being. Our mixed-method pilot study assessed cancer awareness among 478 adolescents in four UK schools using a validated instrument (Cancer Awareness Measure (CAM)) and found that awareness of cancer signs and symptoms was low [19], confirming findings from other small-scale studies conducted in other countries [20-24]. Our study also showed that ‘'Let's talk about it’ statistically significantly increased the number of cancer symptoms adolescents’ recognised, and significantly lowered emotional barriers to help-seeking at the two-week follow-up [16]. However, this pilot study used a quasi-experimental before-and-after design to assess the impact of adolescent cancer education. To our knowledge, the ACE study is the first randomised controlled trial (RCT) to assess the effectiveness of a school-based intervention designed to increase adolescents’ cancer awareness.

Furthermore, our pilot work indicated that some adolescents talked to parents and siblings about cancer following a Teenage Cancer Trust presentation, suggesting that young people may play an important role in raising cancer awareness within families. However, little is known about the role of adolescents as influencers in the upward diffusion of knowledge about cancer to parents/carers. Thus, if our further research confirms that ‘Let’s talk about it’ raises both adolescent and parent cancer awareness, then it may be an important component in future government strategies to detect cancer early [11,12].

### Aim

The aim of the Adolescent Cancer Education (ACE) study is to determine the effectiveness of a school-based educational intervention (that includes components targeted at both adolescents and their parents/carers) to raise adolescents’ cancer awareness (primary outcome), parental cancer awareness and adolescent-parent cancer communication (secondary outcomes). We will determine whether the intervention affects these outcomes in the short-term (two weeks post-intervention) and longer-term (six months post-intervention).

## Methods

### Design

**Figure 1:**
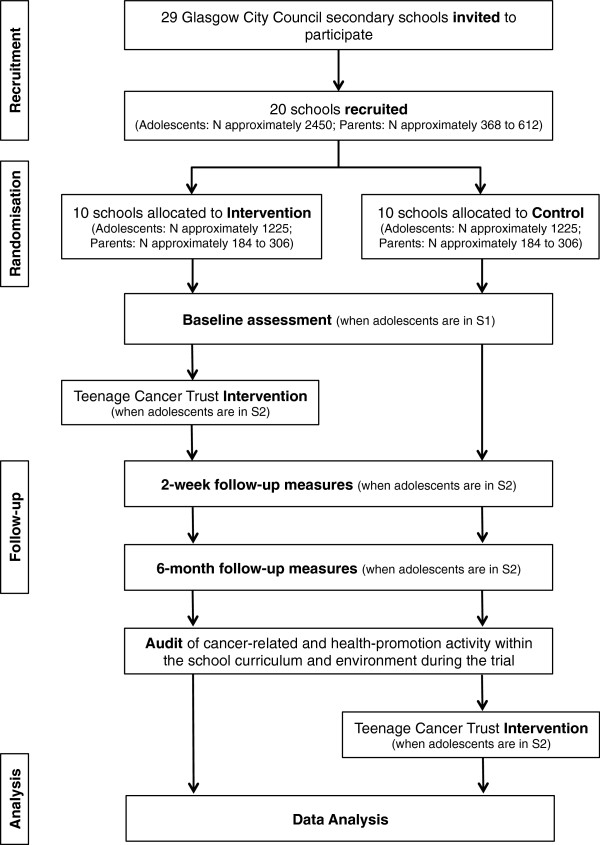
Study flow chart.

ACE is a school-based cluster RCT (Figure 1).

### Setting

All state secondary schools with children in their second year of secondary education (S2) (aged 13 to 14) in the area covered by Glasgow City Council will be invited to participate. Schools are located in areas with different levels of socio-economic deprivation. Special schools (that is, those for adolescents whose additional needs cannot be met in mainstream education) will be excluded because the intervention is not developed for young people with severe learning difficulties and these adolescents may not be able to take part in all of the measurements.

### Participants

Participants will be adolescents in their first year of secondary education (S1) at the point of recruitment and one parent/carer for each student, of the student’s choice (for example, mother, father or foster carer). For adolescents, participation requires attendance at the educational intervention and completion of a homework exercise with their parent/carer in either Autumn 2013 (intervention group) or Spring 2014 (control group), and completion of a self-report questionnaire at baseline, two-week and six-month follow-up. For parents/carers, participation involves completion of a homework exercise with their child in Autumn 2013 (intervention group) or Spring 2014 (control group), and completion of a self-report questionnaire at baseline, two-week and six-month follow-up. Baseline assessment (prior to intervention) will be undertaken with adolescents and parents/carers when adolescents are in the final term of S1. The intervention and two-week and six-month follow-up measures will take place with adolescents and parents/carers when the adolescents are in S2.

#### Sample size calculation

The total number of schools in the research site is 29. We will recruit 20 schools and based on our pilot study response rates (76% of students on school-roll) we will recruit approximately 2,450 adolescents. Our pilot work did not include parents/carers, therefore other school-based research may provide a guide for likely recruitment rates for parents/carers. A previous study examining the influence of a school-based health promotion intervention on parents achieved a 66% response rate [25]. However, given the absence of a precedent for a cancer awareness intervention, we conservatively estimate that we will recruit 15 to 25% of parents, resulting in the inclusion of between 368 and 612 parents.

Sample size calculation is based on this cluster size, with a power of 90% and a two-sided alpha of 0.05. Our previous pilot study showed a difference of 2.7 cancer warning signs recognised between control and intervention group [16]. Assuming an Intraclass Correlation Coefficient (ICC) of 0.05, this study will have a 90% power to detect a difference of 0.5 cancer warning signs between the intervention and control groups. With an ICC of 0.5, this study will still be able to detect a 1.47 difference in the recognition of cancer warning signs.

#### Recruitment and consent

##### Schools

Selection bias will be minimised by inviting all 29 Glasgow City Council mainstream schools to participate in the study and by using an identical recruitment approach for every school. A letter will be drafted by the study researchers and sent by Teenage Cancer Trust to the Headteacher of all schools. The Teenage Cancer Trust educator will follow-up this letter by telephone and/or email to arrange a meeting with either the Headteacher or a teacher identified by the Headteacher with responsibility for health-related curriculum activity or pastoral care. The Teenage Cancer Trust educator and a study researcher will then meet with this identified teacher to discuss and obtain consent for the school’s participation in the study.

##### Adolescents

Parents/carers of all students in S1 will be sent a letter and information sheet about the study which includes a form to be returned to school if they wish to opt their child out of the intervention (that is, Teenage Cancer Trust presentation) and/or outcome assessment. Hence, parents/carers can opt their child out of the outcome assessment but still include them in the intervention. Parents will be given the opportunity to contact the research team to discuss the study by telephone or email.

The opt-out method of parental consent has been found to be an ethical and appropriate way of informing parents and avoids the problems of low response rates and sampling bias encountered in research which has used active consent procedures with parents of young people involved in school-based research [26]. Moreover, the opt-out method was adopted in our previous study and balances the requirement for parental review of their child’s participation with the ethical imperative to minimise selection bias [27].

An information sheet for the adolescent will be given at the time that baseline measurements are undertaken and also at this time they will be asked to give signed consent to their participation in the study. In accordance with best ethical practice, only adolescents who sign the consent form will be included in the study, even if we have parental opt-out consent.

##### Parents/carers

A study information pack containing a letter from the research team, participant information sheet, questionnaire and envelope will be hand-delivered to parents by their child. Parents will be asked to sign the consent form which is appended to the questionnaire at the point they complete the questionnaire. Completed questionnaires will be sealed in the envelope provided and hand-delivered to schools by adolescents.

### Randomisation

We will define recruited schools as high and low deprivation by their score on the Scottish Index of Multiple Deprivation (SIMD) [28]. Due to the skewed deprivation profile of Glasgow, which includes many areas with high levels of deprivation and fewer areas with lower levels of deprivation, SIMD quintile 1 will be coded as high deprivation, with quintiles 2 to 5 as low deprivation to create two groups. Similarly, we will group schools as large (≥150 S1 registered students) or small (<150). We will then group schools by their deprivation and size and randomly allocate them within these strata to intervention or control groups. Randomisation will be undertaken by the trial statistician using SAS 9.3 (SAS Institute, Cary, NC, USA).

#### Blinding

Schools will be informed of their group allocation following randomisation, although this will not be shared with other schools involved in the study. The Teenage Cancer Trust educator will also need to be aware of group allocation in order to schedule school visits; however, this individual will not be involved in data collection or analysis. The trial statistician responsible for randomisation will also conduct data analysis under the supervision of the director of Tayside Clinical Trials Unit (TCTU) at which she is based and alongside a second data analyst based at the University of Stirling who was not involved in randomisation of schools.

### Intervention

Schools randomised to the intervention group will receive the intervention immediately and those randomised to the control group will receive the intervention after the completion of the outcome assessment at six-month follow-up. Thus, the intervention will be delivered in August/September 2013 in those schools randomised to the intervention arm (that is, 6 to 12 weeks following baseline measures), and in April 2014 in those schools randomised to the control arm. Use of a wait-list control group will ensure that all schools receive the intervention. This design has been adopted to minimise drop-out from the trial in the control arm over the six-month follow-up period.

#### Theoretical framework for intervention development

Modifications were made to a previously evaluated intervention [16] to place greater emphasis on increasing adolescents’ communicative self-efficacy based on Bandura’s social cognitive theory [29]. In accordance with social cognitive theory [30] there are four self-efficacy information sources: 1) performance attainments; 2) vicarious experiences; 3) verbal persuasion; and 4) physiological/affective state [31]. Performance attainments involve the individual learning through mastery of specific skills and as a consequence increasing self-efficacy by demonstrating proficiency in a particular behaviour (failure to perform the behaviour may degrade self-efficacy). Vicarious experience refers to the observation of others, who are similar in many respects to the adolescent, successfully performing a particular behaviour and thereby influencing an individual’s own judgement of self-efficacy. Verbal persuasion by others who are knowledgeable about a particular behaviour is designed to increase an individual’s sense of ability and skill to perform a particular task and, hence, their self-efficacy. It refers to others expressing faith in the skills of the individual to perform the behaviour, thereby enhancing that individual’s perception of efficacy. Physiological and affective states are also reported to influence self-efficacy. Table 1 shows how the intervention was adapted using social cognitive theory to improve adolescent’s communicative self-efficacy.

**Table 1:** Improving communicative self-efficacy

**Information source**	**Change techniques**
1. Performance attainments: mastering the skill of cancer communication	Homework to enhance family communication about cancer.
2. Vicarious experience: exposure to young people of a similar age who have mastered the task of cancer communication.	Video clips of young people talking about cancer.
3. Verbal persuasion: exposure to an empathetic and knowledgeable educator	Video clips explaining why it is good to talk to parents about your health.
4. Physiological and affective states	Video clips addressing worries and anxieties associated with help-seeking.

#### Intervention content and delivery

The modified intervention comprises two key components:

1) a one-hour verbal and visual presentation delivered by a Teenage Cancer Trust educator to adolescents in school; and

2) provision of a parent-adolescent homework activity.

The same Teenage Cancer Trust educator will deliver the presentation. She has 3.5 years’ experience of delivering the intervention and has previous experience in health promotion. She will deliver the presentation to a whole year group on the school premises (for example, large hall) during school time. The audience will include all students whose parents have not opted their child out of receiving the intervention. Thus, the audience may include students whose parents have opted them out of the study, but not the intervention. The presentation is 50 to 60 minutes in duration with variation within this timeframe to accommodate differences in the school timetable. The presentation encourages interaction between the educator and students through the use of a true or false quiz and open questions to the audience. The educator will also utilise video clips to inform adolescents about: cancer signs, symptoms, diagnosis and treatment of both TYA cancers, and those more common in later adulthood; their emotional impact; and how young people can reduce their risk of developing cancer in the future. The same presentation format will be followed in all schools and is summarised in Table 2.

**Table 2:** Presentation description

**Learning objective**	**Technique**	**Time (minutes)**
Introduction	Verbal information on what the session is going to cover and allow people to leave if they feel uncomfortable	2
Encourage open discussion about cancer	Role play – young people act out a scenario with the person sitting next to them and feed back to the speaker	3
Encourage open discussion about cancer	True or false quiz with students conferring on the answers	5
Encourage open discussion about cancer	Film clip of talking openly about cancer and explaining why it is important to talk about it	2
What is cancer	Verbal and written information on basic biology of cancer, with pictures of normal and abnormal cells	5
Explanation of why cancer information is important for this age group	Verbal and written information on numbers of young people, and general population, with cancer in the UK and emotions involved with a cancer diagnosis	5
Issues around delays in diagnosis in young people with cancer	Verbal ‘story telling’ of real life case study; film clip	6
Signs and symptoms of cancer	Film clips of young people describing their symptoms; verbal and written description	5
Types of cancer	Written list and verbal description	5
Information about ways in which to reduce the risk of developing cancer later in life	True or false quiz about: Smoking, alcohol, diet, exercise and sun safety	10
How cancer is treated and side effects of treatment	Verbal information on chemotherapy, radiotherapy and surgery	5
Information about Teenage Cancer Trust	Film and verbal information about what the charity does to help young people with cancer	5
Recap key facts and challenge young people to tell family what they have learned	Parent-adolescent homework activity sheet	2

The parental component comprises a homework sheet given to students at the end of the presentation to take home and complete with a parent/carer. The students will be encouraged to ask their parent/carer six questions relating to cancer and have a discussion with them around these questions. This is designed to encourage conversations about the Teenage Cancer Trust presentation which may subsequently raise parents’ cancer awareness.

To assess completion of this homework activity a proforma will be emailed by the Teenage Cancer Trust educator to class teachers at two-week follow-up which will request information on the percentage of distributed homework activity sheets adolescents have returned to school completed. This will provide contextual data to understand potential differences in intervention effectiveness between schools in terms of adolescent-parent cancer communication.

### Data collection

Adolescent and parent outcomes will be assessed using a self-report questionnaire at three time points:

1) Baseline (T_0_): June 2013; when adolescents are in school year S1.

2) Two-week follow-up (T_1_): August/September 2013; when adolescents are in the first term of S2 and approximately two weeks after the intervention.

3) Six-month follow-up (T_2_): March 2014; when adolescents are in the second term of S2.

Our pilot study reported that 88% of students who completed baseline assessment completed two-week follow-up, and 77% completed six-month follow-up [16]. However, loss to follow-up at six months was increased in our pilot study by the inclusion of senior school students who were participating in final examinations at the time of outcome assessment. The younger age group included in the ACE study mitigates against this limitation of our pilot work. Moreover, modifications have been made to the consent form to increase the legibility of students’ names used to link data across data collection time points. Thus, for these reasons we anticipate loss to follow-up to be lower than in our previous study.

Table 3 outlines the outcome measures, mediators and unintended consequences that will be assessed for adolescents and parents/carers at each of the three data collection time-points. All measures will be combined into single and separate self-completion paper questionnaires for adolescents and parents/carers that will be the same for each round of data collection.

**Table 3:** Measures assessed at each data collection time point

	**Adolescents**			**Parents/Carers**		
Measures	T_0_	T_1_	T_2_	T_0_	T_1_	T_2_
*Outcomes*						
Cancer Awareness Measure (CAM)	*√*	*√*	*√*	*√*	*√*	*√*
Adolescent-parent cancer communication	*√*	*√*	*√*	*√*	*√*	*√*
*Mediators*						
Communicative self-efficacy	*√*	*√*	*√*	-	-	-
Family Communication Scale (FCS)	*√*	*√*	*√*	*√*	*√*	*√*
Cancer risk perception	*√*	*√*	*√*	-	-	-
*Unintended consequences*						
Hospital Anxiety and Depression Scale (HADS)	*√*	*√*	*√*	-	-	-

Adolescent questionnaires will be administered by teachers in the classroom. Teachers will be available to answer any queries and to assist the students with reading and writing as necessary. Students will be asked to complete the questionnaire in complete silence but will be informed that it is not a test. Parent/carer questionnaires will be sent home from and returned to school with adolescents. Study researchers will be available by telephone and email to answer any queries parents may have when completing the questionnaire. Specific measures to assess outcomes, mediators and unintended consequences included in the questionnaire are described in detail below.

#### Outcome measures

The primary outcome is adolescent cancer awareness measured two weeks after the intervention. Cancer awareness will be assessed using items from the Cancer Awareness Measure (CAM), details of which are published elsewhere [19,32]. The CAM comprises nine questions to measure awareness of warning signs of cancer, cancer help-seeking, cancer risk factors, common cancers and screening programmes. Questions about awareness of screening programmes are omitted from the adolescent questionnaire as these are not directly relevant to this age group.

Secondary outcomes that will be assessed are:

1) Parent/carer cancer awareness using the nine questions in the CAM (that is, no CAM items have been omitted from the parent/carer questionnaire);

2) Adolescent-parent cancer communication using two questions adapted from our pilot study. Adolescents will be asked if they have spoken to their mother, father or someone else about cancer in the previous two weeks, and, if so, to indicate from a list of topics what they had talked about (for example, warning signs of cancer, cancer risk factors, help-seeking, cancer screening, common cancers). Parents will also be asked whether they spoke to their mother, father, partner/spouse, child or someone else about cancer and will also be asked to indicate the topic of conversation.

#### Mediators

Increasing our understanding of the mechanisms of change in cancer awareness is essential for designing and delivering more effective interventions to raise public cancer awareness. Based on social cognitive theory [29] (that is, Health Belief Model (HBM) [33,34] and Health Action Process Approach (HAPA) [35]) the following mediators of change in adolescent and parent/carer cancer awareness and communication will be assessed:

1) Adolescent communicative self-efficacy using a six-item scale, with a 5-point Likert scale to indicate the extent of their agreement with the items;

2) Family communication using the The Family Communication Scale (FCS) [36,37], a 10-item instrument with a 5-point Likert scale that measures the degree of openness in family communication.

3) Adolescent cancer risk perception using four questions with a 5-point Likert scale: 1) If I do not have a healthy lifestyle, my chances of getting cancer at some point in my life are: very small/small/neither big or small/big/very big; 2) If I have a healthy lifestyle my chances of getting cancer at some point in my life are smaller: completely disagree/disagree/neither agree or disagree/agree/completely agree; 3) If I do not have a healthy lifestyle, I feel: very vulnerable/vulnerable/a little vulnerable/not vulnerable/definitely not vulnerable to getting cancer at some point in my life; 4) If I have a healthy lifestyle, I feel less vulnerable to getting cancer at some point in my life: completely disagree/disagree/neither agree or disagree/agree/completely agree.

These mediators are derived from both theoretical and empirical work on communication in the context of parent-adolescent relationships, and the importance of risk perception as a key construct in the HBM and HAPA. Communication has been defined as the assertive and inoffensive expression of ideas and feelings and the attentive and accurate receiving of ideas expressed by others [38]; open communication refers to the ability to share feelings, approach difficult topics and ask for help [39]. Parent-adolescent communication has been shown to influence adolescent health-risk behaviours, most notably sexual risk behaviour [40-47] and a recent systematic review of 12 interventions for improving parental communication about sex with adolescents aged 11 to 18 years reported communication skills as most effective for transmitting sexual health knowledge and decision-making skills to their adolescents [48]. Risk perception has been studied as an outcome variable in cancer risk communication efforts [49].

#### Unintended consequences

One potential unintended consequence of adolescent cancer education may be heightened anxiety. Anxiety will, therefore, be assessed using the Hospital Anxiety and Depression Scale (HADS) [50], which has been validated with adults [51] and adolescents [52] and used in school-based studies [53,54].

### Statistical analysis

The presentation of findings from the trial will be in accordance with CONSORT (Consolidated Standards of Reporting Trials) guidelines for cluster RCTs [55].

Data will be analysed using SAS 9.3 (SAS Institute, Cary, NC, USA). Descriptive statistics will be calculated for all data and reported as mean (SD) for continuous data and N (%) for categorical data. Data will be tested for normality and transformations of the outcome variables will be used where necessary. Differences between the intervention group and control group for the outcome measures will be tested using multiple regression methods, adjusted for clustering by school. Changes from baseline to post intervention will be assessed with baseline parameters as covariates, taking into account the repeat measures design. A detailed statistical analysis plan will be created and finalised before analysis is performed.

### Audits evaluation

Two audits will be conducted to assess:

1) Cancer-related and health promotion activity within the school curriculum and environment;

2) Fidelity of intervention content and delivery.

An audit will be undertaken in all schools, in both the intervention and control arms, to identify any issues that might impact effectiveness. The audit will take place after six-month follow-up measures have been completed and will gather information about the following:

1) Cancer-related concerns and activities undertaken by the school (for example, fund-raising, staff/student cancer diagnosis or bereavement, S2 curriculum topics);

2) Health promotion activities conducted within the school (for example, fund-raising, awareness raising, S2 curriculum topics).

The main contact member of staff for the ACE study in each school will be invited to participate in a face-to-face semi-structured interview to assess cancer-related and health promotion activities and concerns in the school within the previous 12 months (that is, during the ACE study).

An audit will also be undertaken to ascertain whether the intervention was delivered as planned. The Teenage Cancer Trust educator will keep an intervention log that will record the date that the presentation was delivered in all schools (intervention and control arms) and report, and provide a rationale for, deviations in intervention delivery from protocol (see Table 2). This log will be reviewed by study researchers to assess potential impacts of any changes to intervention delivery on differences in intervention effectiveness between schools.

### Data management and deposition

All personal information (including school names, student and parent/carer names, and contact details) will be stored electronically and in paper form in a secure password-protected/securely locked filing cabinet on University computer hardware/premises. Information on individual adolescent’s measurements will not be disclosed to teachers or anyone else; anonymised data will be available to schools upon request for further analysis or use in teaching.

In accordance with the conditions of use of the CAM, data collected for the purposes of this study will be deposited in the UK Data Archive. Data subsequently made available to researchers through this archive will be anonymised and individual participants will not be identified.

### Ethical approval

The ACE study was reviewed and approved by the Research Ethics Committee in the School of Nursing, Midwifery and Health at the University of Stirling.

## Discussion

This paper outlines a protocol for a cluster RCT (ACE) that aims to determine the effectiveness of a school-based educational intervention designed to raise adolescents’ and parents’ cancer awareness and cancer communication within families. Schools are established loci for health promotion as adolescents can be easily reached through schools [56,57]. Thus, schools may be a useful arena for raising adolescents’ cancer awareness, and, through adolescents’ role as influencers, diffusion of cancer knowledge to parents and increased cancer communication within families. However, there are few school-based programmes designed to raise cancer awareness [58] and, to our knowledge, none that have been evaluated using a cluster RCT design.

The ACE study addresses a number of limitations of our previous pilot work of a school-based cancer awareness raising intervention [16], most notably: 1) intervention development drawing on social cognitive theory; and 2) use of a more robust experimental design. Our pilot study evaluated an existing educational intervention and adopted a quasi-experimental before-and-after design. ACE will develop a theoretically informed intervention and use a more robust cluster RCT design to assess effectiveness.

Complex educational interventions might not be easily generalized from one school or country to another due to the important influence of contextual factors [59]. However, if found effective, ACE has potential to provide evidence to inform the development of on-going public cancer awareness initiatives in the UK, such as Detect Cancer Early (DCE) in Scotland and the National Awareness and Early Diagnosis Initiative (NAEDI) in England, as well as efforts elsewhere internationally to increase adolescent and parent cancer awareness and family cancer communication.

## Trial status (16 August 2013)

We have obtained ethics approval and funding for the study from Teenage Cancer Trust and Scottish Government Detect Cancer Early Programme. We recruited 20 out of 29 schools in the area covered by Glasgow City Council in May 2013. Two schools responded and indicated that they would not participate and seven schools were unable to be reached by telephone in the time given to recruit schools. The 20 schools that have consented to take part include 9 schools in SIMD 1 (most deprived) and 11 schools are in SIMD 2 to 5; 12 schools have ≥150, S1 registered students. There are 3,223 eligible students on the S1 school roll in the 20 recruited schools. Baseline data collection has been conducted in all 20 schools with adolescents and parents/carers.

## Abbreviations

ACE: Adolescent cancer education; CAM: Cancer awareness measure; CONSORT: Consolidated standards of reporting trials; DCE: Detect cancer early; FCS: Family communication scale; GP: General practitioner; HAPA: Health action process approach; HADS: Hospital anxiety and depression scale; HBM: Health belief model; ICC: Intraclass correlation coefficient; RCT: Randomised controlled trial; TYA: Teenage and young adult; NAEDI: National awareness and early diagnosis initiative; PSHEE: Personal, social, health and economic education; TCTU: Tayside Clinical Trials Unit; S1: Secondary 1; S2: Secondary 2; SD: Standard deviation; SIMD: Scottish index of multiple deprivation.

## Competing interests

The authors declare that they have no competing interests.

## Authors’ contributions

RGK, GH and LF completed all phases of pilot work for ACE. RGK, IM, GH and RDN modified the intervention. GH and IM were responsible for participant recruitment and managed data collection, while PR conducted sample size calculations, randomisation and will be responsible for data analysis. RGK managed data entry and will conduct data analysis. RGK, RO’C, RDN, LF, SH and GH contributed to the design of the ACE study. GH secured funding and ethical approval and RGK and GH wrote the first draft of this protocol. All authors contributed to the final version of this protocol and will be responsible for conducting the ACE study. All authors read and approved the final manuscript.
